# An electrophysiological and proteomics roadmap for human induced glutamatergic neurons: fine-tuning of culture conditions for pathophysiological studies

**DOI:** 10.1038/s41420-026-03185-w

**Published:** 2026-06-05

**Authors:** Martina Servetti, Giulia Parodi, Martino Caramia, Ennio Nano, Martina Bartolucci, Antonella Marte, Giacomo Mazzoni, Simone Giubbolini, Farah Diab, Andrea Petretto, Pierluigi Valente, Sergio Martinoia, Simona Baldassari, Anna Fassio, Fabio Benfenati, Anna Corradi, Bruno Sterlini

**Affiliations:** 1 Dipartimento di Medicina Sperimentale, Università di Genova, Genoa, Italy; 2 Center for Synaptic Neuroscience and Technology, Istituto Italiano di Tecnologia, Genoa, Italy; 3 IRCCS Azienda Ospedaliera Metropolitana, Genoa, Italy; 4 Core Facility for Omics Science, IRCCS Istituto Giannina Gaslini, Genoa, Italy; 5 Department of Informatics, Bioengineering, Robotics, and Systems Engineering (DIBRIS), University of Genova, Genoa, Italy; 6 Unit of Medical Genetics, IRCCS Istituto Giannina Gaslini, Genoa, Italy; 7 Department of Neurosciences, Rehabilitation, Ophthalmology, Genetics, Maternal and Child Health (DiNOGMI), University of Genova, Genoa, Italy

**Keywords:** Development of the nervous system, Stem-cell differentiation, Neurophysiology

## Abstract

Induced glutamatergic neurons (iGluNeurons) generated by Neurogenin-2 (NGN2) overexpression in human pluripotent stem cells are a powerful model for studying human neuronal maturation and function; however, NGN2-based protocols still lack standardized culture conditions that critically affect neuronal development and function. Three key factors have been identified by previous literature, namely the composition of extracellular matrix coating, the initial plating density, and the choice of culture medium, but the differential effects of their combination have not been thoroughly analyzed. Here, we investigated the combinatorial effects of these three variables, testing eight distinct culture conditions resulting from the combinations of two coatings (poly-L-ornithine and polyethyleneimine), two media (BrainPhys and Neurobasal), and two cell densities (4800 and 1200 cells/mm²). We assessed electrophysiological properties at the single-cell and network levels, characterized morphofunctional and proteomic features across multiple developmental stages. Electrophysiological data indicate that medium composition and plating density, rather than substrate coating, determine neuronal maturation dynamics, with BrainPhys and high density promoting rapid but transient maturation while Neurobasal and low density supporting gradual and sustained network development. Morphofunctional analyzes of synapses and the axon initial segment, together with neuronal maturation markers, support an early BrainPhys-driven acceleration of development that is later exceeded by Neurobasal. To enable accurate proteome profiling of the iGluNeuron system—comprising human neurons and rat astrocytes—we developed a robust taxonomic filtering algorithm that selectively identifies human-specific proteins. This approach confirmed the presence of a conserved core of NGN2-driven differentiation pathways across all settings, in addition to condition-specific signatures. Finally, in the optimal conditions identified through our experimental analyzes, robust spontaneous and evoked synaptic activity was observed. These results provide a framework for optimizing iGluNeuron cultures, balancing rapid maturation and long-term functional stability, and establishing a benchmark for human neuronal models in disease research and drug screening.

## Introduction

Induced neurons (iNeurons) generated via the forced expression of Neurogenin-2 (NGN2) in human pluripotent stem cells (hPSC) represent a highly efficient and robust model system for studying human neuron maturation and function. NGN2-mediated differentiation allows for the rapid conversion of hPSCs or fibroblasts into functionally mature excitatory neurons (iGluNeurons) within a remarkably short timeframe [1]. This approach bypasses the need for complex, multi-step differentiation protocols and overcomes many of the limitations associated with extrinsic factor-based differentiation, such as variability in neuronal subtype specification and low yield [2, 3]. As a result, iGluNeurons have gained widespread adoption in neurodevelopmental and neurodegenerative research, particularly for disease modeling, drug screening, and potential therapeutic applications.

Despite its widespread use, standardization of culture conditions remains a major challenge. A systematic review of the literature, across 54 peer-reviewed studies, revealed that current NGN2-based differentiation protocols exhibit several differences that can introduce substantial variability in neuronal maturation, network formation, and functional output [3–56]. Such inconsistencies hinder cross-study comparisons and limit result reproducibility, thereby affecting the interpretation of experimental outcomes. Since induced neuronal cultures are frequently used for pathophysiological studies and high-throughput drug screening, the establishment of optimized and standardized culture conditions is imperative. Among the various culture parameters, three factors appear to have a key role in determining the efficiency of neuronal differentiation and functional maturation: (1) extracellular matrix coating, (2) initial plating density, and (3) cell medium. Differences in these parameters can significantly influence neuronal maturation and electrophysiological properties, and thereby the interpretation of the experimental results.

To address this issue, we conducted a systematic comparison of eight distinct combinations of coating, cell density and medium conditions, evaluating their effects on neuronal morphology, proteomic profile, development, and functional maturation. We found that, while substrate coating had minimal impact, both medium composition and plating density strongly influenced the maturation dynamics and neuronal activity. The standardized combinations of culture conditions enable a balance between rapid maturation and long-term functional stability that can be useful to highlight specific disease-related phenotypes. These results provide a benchmark for the design of human neuronal models for brain diseases and high-throughput drug screening.

## Results

### Systematic review of culturing conditions for induced glutamatergic neurons

We performed a systematic review of the literature related to NGN2-mediated differentiation to glutamatergic neurons (see “Materials and methods” section for the criteria applied during selection). We selected 54 peer-reviewed papers and found high variability across the culture conditions for generating glutamatergic neurons (see Table 1). Based on the results of this analysis, the culture conditions that across all protocols appeared to be the most critical — while also showing the greatest variability — are: culture media, coating substrates, and cellular density.

**Table 1:** List of published papers analyzed in a systematic review of NGN2-iGLuNeuron.

Author	Date	Neuronal induction	Replating	Coating /conc	Media	N° of cells/support	Cell Density calculation (cells/mm^2^)	Ref
Zhang et al.	2013	NGN2	No	Matrigel	NB	15,000 cells/ 24-well plate	81	[3]
Rubio et al.	2016	NGN2 /ASCL1 / DUAL SMAD	Yes	ND	DM	100,000 cells coverslip (ND)	150	[4]
						600,000 cells/ MEA plates (ND)	306	
Lam et al.	2017	NGN2	No	100 ug/ml PDL	NB A	63,000 cells/ 14 mm coverslips	412	[5]
Frega et al.	2017	NGN2	No	50 ug/ml PLO, 20 ug/ml ML	NB	75,000 cells/ 6-wells MEA plate	2344	[6]
						20,000 cells/ Coverslips for 24-well plate	107	
Russel et al.	2018	NGN2	No	Matrigel	NB	ND	ND	[7]
Fernandopulle et al.	2018	NGN2	Yes	PLO or PEI, 10ug/ml LM	DM or NB A or BP	10,000–50,000 cells/ 96-well plate (imaging)	439	[8]
						30,000–150,000 cells/ Ibidi μ-slide 8-well slide (imaging)	3000-1500	
						10,000 cells/ 10 cm dish (biochemistry)	24	
						1500 cells/ 6-well plate (biochemistry)	17	
Meijer et al.	2019	NGN2	Yes	PLO, LM	NB	50,000 cells/cm^2^	500	[9]
Schafer et al.	2019	NGN2	No	10 ug/ml PLO, 5ug/ml ML	NB	ND	ND	[10]
Rhee et al.	2019	NGN2 / ASCL1-DLX2	Autaptic	PDL, collagen	NB	From 3500 to 8000 cells per 100 mm^2^ in micro-island astrocyte.	35 80	[11]
Peitz et al.	2020	NGN2 /ASCL1-DLX2	Yes	15 mg/ml PLO, ML	DM	100,000 cells/ 96 well plates	292	[12]
Rosa et al.	2020	NGN2	No	PLO, ML	BP	80,000 cells/ 24-well plates	800	[13]
Ordureau et al.	2021	NGN2	No	Matrigel	NB	30,000 cells/cm^2^ 35 mmFluoroDishes	300	[14]
Schörnig et al.	2021	NGN2	No (cited Frega et al.)	50 mg/ml PLO, 20 mg/ml ML	NB	ND	ND	[15]
Lin et al.	2021	NGN2	No	50 mg/ml PLO, 10 mg/ml ML	NB	ND (cited Frega et al.)	ND	[16]
Mossink et al.	2021	NGN2	No	50 mg/ml PLO, 5 mg/ml HL or 20 μg/ml ML	NB	20,000 cells/ MEA chip	600	[17]
Song et al.	2021	NGN2/ ASCL1 and DLX2	No	PEI, ML	BP	130,000/cm^2^ 12-well plate;	1300	[18]
						260,000 cells/ 12-well plate	751	
Shih et al.	2021	NGN2	Yes	ND	N2B27 (50% DM, 50% NB)	15,000–25,000 cells/cm2 (48-well MEA plates)	150-250	[19]
Li et al.	2021	NGN2	No	Matrigel	BP	17,000 cells/ 48-well MEA plate	531	[20]
Gunnewiek et al.	2021	NGN2	No	50 μg/ml PLO, 5 μg/ml HL	NB	20,000 cells/ 24-well MEA plate	625	[21]
Schmid et al.	2021	NGN2	No	ND	NB	10,000 cells/ 24 well plate	313	[22]
Galiakberova et al.	2021	NGN2	Yes	BD-Matrigel	N2B27 (NB, DM)	30,000 cells/cm^2^ 35 mm culture dishes	300	[23]
Muzzi et al.	2021	NGN2	Yes	1% chitosan	NB	2500 cells/ mm2	∼2500	[24]
Wang et al.	2021	NGN2	No	Matrigel	BP	400,000–600,000 24-well plate	1445	[25]
Strauß et al.	2021	NGN2	No	100 μg/ml PLO, 10 μg/ml ML	N2B27	ND	ND	[26]
Sanchez et al.	2021	NGN2	Yes	PDL, ML	DM	75,000 cells/ 96-well plate	2193	[27]
Cheng et al.	2021	NGN2	Yes	20 µg/m PLO, 5 µg/m ML	NB 48%, DM 48%	300,000 cells/ 24-well plates	867	[28]
						800,000 cells/ 6-well plates	886	
Lee et al.	2022	NGN2	No	PEI (0.1%), 10 µg/mL ML	BP	~165,000 cells/ 12-well plates	477	[29]
Girardin et al.	2022	NGN2	Yes	PDL, ML	NB	30,000 to 65,000 cells/ cm2	300 to 650	[30]
Wen et al.	2022	NGN2 or ASCL1/DLX2	Yes	0.07% PEI, 3.3 µg/ml ML	NB	500/mm^2^ and 1000/mm^2^	500 and 1000	[31]
McSweeney et al.	2022	NGN2	Yes	Matrigel	NB	200,000 cells/ 24-well plates	578	[32]
						300,000 cells/ HD-MEA (50 μL drop)	4545	
Griffin et al.	2023	NGN2	Yes	Matrigel	NB A	20.000 cells/ 10 cm petri dish	3328	[33]
Wen et al.	2023	NGN2	Yes	Matrigel	NB	60,000 cells/ 24-well MEA plate	1875	[34]
						100,000 cells/ 6-well plate	1108	
Feuer et al.	2023	NGN2	Yes	PLO (0.01%), ML	NB	150	888	[35]
Faheem et al.	2023	NGN2	No	Matrigel	NB	500,000 cells/ 6-well plate	55	[36]
Wang et al.	2023	NGN2 / ASCL1	Yes	PLO (50 μg/mL), HL (5 μg/mL)	NB	18,000 cells/ 24-well MEA plate	180	[37]
van Hugte et al.	2023	NGN2	No	PLO (50 μg/ml), HL (5 μg/ml)	NB	20,000 cells/ 24-well MEA plate	200	[38]
Supakul et al.	2023	NGN2	No	1 μg/mL PLL 3.0 μg/ml iMatrix-511 silk	NB Plus	50,000 cells/ 24-well plate	1563	[39]
Pham et al.	2023	NGN2	Yes	100 μg/m PLO, Matrigel	NB	ND (cited Schafer et al.)	ND	[40]
Bullmann et al.	2024	NGN2	Yes	PDL, Matrigel	NB	72,000 cells/ 24-well plates (25 ul drop)	1756	[41]
Mamais et al.	2024	NGN2	Yes	Geltrex	NB	120,000 cells/ 12 mm coverslips	9	[42]
Das et al.	2024	NGN2	No	Matrigel	NB A	100,000 cells/ 24-well plates	537	[43]
Heider et al.	2024	NGN2 / ASCL1	Yes	PLO, ML	NB Plus	125,000 cells/cm2	1250	[44]
Glass et al.	2024	NGN2	No	Geltrex	ND(cited Zhang et al., 2013)	ND		[45]
Maksour et al.	2024	NGN2	No	10 μg/ml PDL ML	BP	10,000 cells/cm2	100	[46]
Ma et al.	2024	NGN2	No	ND	ND	ND		[47]
Dhaliwal et al.	2024	NGN2	Yes	PEI	Neurocell medium	50,000–100,000 cells/ 24-well-plate	268 54	[48]
Qu et al.	2024	NGN2	Yes	Matrigel	BP	50,000 cells/ 24-well plate	268	[49]
Dou et al.	2024	NGN2	Yes	PLO	BP	300,000 cells per dish	50	[50]
Parodi et al.	2024	NGN2 / ASCL1	Yes	50 µg/ml PLO, 20 µg/ml HL	NB	1200 cells/mm^2^	1200	[56]
Villegas et al.	2024	NGN2	No	Matrigel	NB	100,000 cells/ cm^2^	1000	[52]
Lemieux et al.	2024	NGN2	Yes	0.1 mg/mL PDL, 20 μg/mL ML	BP	90,000 NPC/ 24-well MEA plate	5400	[53]
Shan et al.	2024	NGN2	Yes	50 μg/ml PDL, iMatrix	NB or NB A, NB Plus or BP or iCell Medium 1	750,000 cells/ 6-well MEA plates (50 μL drop)	7500	[54]
Sébastien et al.	2025	NGN2	Yes	10 µg/ml PLO, 5 µg/ml ML	BP	15,000 cells/cm^2^	150	[55]
NeuCyte company	/	NGN2 / ASCL1	Yes	PEI, ML	ND	270,000 cells/ 100 mm^2^	2700	
BitBio company	/	NGN2	Yes	/	/	30,000 cells/cm²	300	

In the column labeled “Cell Density Calculation,” all cell densities were converted to cells/mm² to enable cross-study comparison. When density values were not explicitly reported, but seeding numbers and culture surface areas were available, the final density was estimated based on this information.

*NB* neurobasal, *BP* BrainPhys, *ML* Mouse Laminin, *HL* Human Laminin, *L* Laminin, *DM* DMEM/F12, *PLO* poly-L-ornithine, *PDL* poly-D-lysine *ND* not defined.

The most commonly used media were Neurobasal (61%, including all variants: standard Neurobasal, Neurobasal Plus, and Neurobasal A), BrainPhys (20%), and DMEM/F12 (14%, accounting for both mixed-media preparations combining Neurobasal and DMEM/F12 as well as DMEM/F12 alone). The remaining 5% included unspecified or insufficiently described media. Based on these findings, we selected Neurobasal (NB) and BrainPhys (BP) media for our experiments.

Most of the studies employed a double-coating approach (62%), typically combining poly-ornithine or poly-lysine with Matrigel or laminin (of either human or murine origin). The remaining 38% used a single-coating method. Specifically, the most prevalent double-coating combination was poly-L-ornithine (PLO) and laminin (32.7%), followed by polyethyleneimine (PEI) and laminin (9.1%). Among single-coating protocols, Matrigel was the most frequently used (23.6%) since it is commonly employed to plate and culture hiPSCs for direct differentiation. However, since our protocol (schematized in supplementary Fig. 1A) includes a replating step and aims to enhance cell differentiation, we prioritized double-coating strategies by selecting PLO/laminin and PEI/laminin for testing.

Our analysis of cell density revealed that the two most common ranges are below 1100 cells/mm² (73.2%) and above 1500 cells/mm² (17.9%), with 10 papers lacking clear cell density specifications. It is important to note that the cellular densities used vary depending on the experimental setting. As an example, for immunostaining the densities typically range from 100 to 1000 cells/mm², depending on the substrate. In contrast, for MEA-based analysis of neuronal activity, two optimal plating densities have been identified for comparison: 1200 cells/mm² and 4800 cells/mm^2^ [6, 32].

In summary, based on the systematic review analysis of published studies on iGluNeurons generated using the NGN2 overexpression, we selected the following conditions for functional outcome comparison: (i) *medium*: NB and BP; (ii) *cellular density*: 1200 cells/mm² (low density, LD) and 4800 cells/mm² (high density, HD); and (iii) *coating*: PLO and PEI. As shown in supplementary Fig. 1B, we obtained a matrix of eight conditions to evaluate the impact of these three variables at the functional level.

### PEI and PLO adhesion factors did not affect the neuronal networks’ electrophysiology

**Fig. 1:**
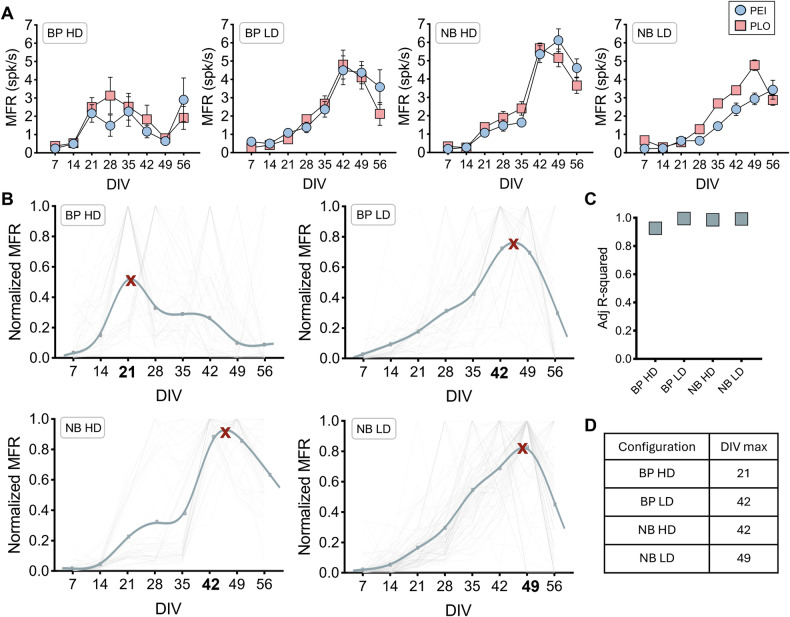
Effects of surface coating with adhesion molecules. **A** Mean Firing Rate (MFR) trend during the development of the neuronal cultures cultured on PEI- (light blue) or PLO- (pink) treated surface of the BP-HD, BP-LD, NB-HD, and NB-LD conditions. Data are represented with the mean (dot) and the standard error of the mean (whiskers). **B** Normalized MFR trend of each BP-HD (top left), BP-LD (top right), NB-HD (bottom left), and NB-LD (bottom right) cultures (light grey) with the fitting of the average MFR overimpressed in dark grey. The mean MFR per DIV is reported with a square. The maximum of the profile is highlighted with a red cross, and the closest DIV in which the recordings were performed is reported in bold on the *x*-axis. **C** Adjusted *R*-squared of the normalized MFR profile fitting for each condition. **D** Table with the DIVs in which the networks reached the maximum of the MFR profile for each condition. Data are shown as means ± SEM. Data were obtained as described in the MEA recordings and data analysis section. All *p*-values of the computed statistical comparisons, including the significant ones, are reported in the Supplementary Table 1A.

We first evaluated the development of the neuronal cultures to observe both changes related to the different adhesion factors and the evolution of the firing trend. Interestingly, PEI and PLO coating led to similar development profiles along all the conditions without statistical differences (Fig. 1A). For this reason, we decided to merge the dataset with the same culture medium and density obtained from PEI and PLO. We then computed the normalized mean firing rate (MFR) from the merged dataset to identify the peak of neuronal activity (DIV max) during culture development (Fig. 1B). Reasonably, the BrainPhys high-density configuration (BP-HD) showed a maximum at DIV 21, while the same medium at low density (BP-LD) led to a shift in the maturation of the neuronal cultures (DIV max = 42) comparable to the Neurobasal High Density (NB-HD, DIV max = 42). Finally, the slowest development of the neuronal cultures was shown in the Neurobasal low-density configuration (NB-LD), in which the maturation was reached at DIV 49. We chose to carry out the direct comparison among these 4 configurations (see Section Results: “Electrophysiological signatures of the four culture conditions with highest spontaneous firing”) in the DIV max of each of them to have the same stage of the neuronal cultures’ development. Finally, to strengthen this analysis and prove the validity of our used firing trend fitting, we computed the adjusted R-squared (Fig. 1C) which showed values between 0.9261 (BP-HD) and 0.9948 (BP-LD), demonstrating a high accuracy. The summary of the found DIV max for each configuration is reported in Fig. 1D.

### Low- and high- density-related shift in the maturation of the networks

**Fig. 2:**
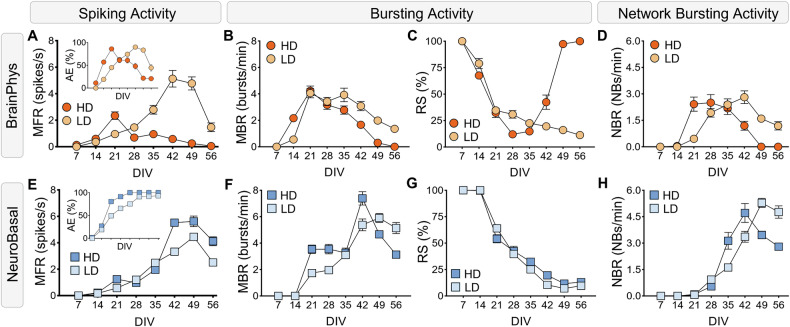
Effects of the cellular density. Trend during the development for BP-HD (red) and BP-LD (orange) cultures of **A** Mean Firing Rate (MFR) and percentage of Active Electrodes (AE, inset); **B** Mean Bursting Rate (MBR); **C** Percentage of Random Spikes (RS); **D** Network Bursting Rate (NBR). Trend during the development for NB-HD (blue) and NB-LD (light blue) cultures of **E** Mean Firing Rate (MFR) and Percentage of Active Electrodes (AE, inset); **F** Mean Bursting Rate (MBR); **G** Percentage of Random Spikes (RS); **H** Network Bursting Rate (NBR). Data are shown as means ± SEM. Data were obtained as described in the MEA recordings and data analysis section. All *p*-values of the computed statistical comparisons, including the significant ones, are reported in the Supplementary Table 1A.

To evaluate the differences between the low- and high-density cultures, we considered the culture media separately, by analyzing first the electrophysiological features of the BP networks (Fig. 2A–D) and, subsequently, those of the NB ones (Fig. 2E–H). Concerning the BP cultured networks, the percentage of active electrodes (AE) reflected the development of the firing rate, reaching the maximum at DIV21 for HD and DIV42 for LD cultures. After the peak in the development, both the MFR (Fig. 2A) and the percentage of the AE (Fig. 2A inset) decreased, proving a decay of the networks. Regarding the bursting activity, the different densities highlighted a delayed insurgence of burst events in the LD cultures (Fig. 2B), while the percentage of random spikes (RS, Fig. 2C) decreased over time for both configurations, indicating progressive network maturation and the emergence of more synchronous firing patterns. Despite this, the HD cultures after DIV35 revealed an RS increase reaching 100% of random activity in the last recorded temporal step, proving a complete regression of the networks, which was also reflected in the network bursting rate (NBR, Fig. 2D). Indeed, the high-density NBR increased during the first DIVs, reaching its maximum around DIV 21 and, subsequently, approaching values equal to zero at DIV 49 and DIV 56.

On the other hand, the NB neuronal cultures, at both LD and HD, demonstrated an increasing percentage of active electrodes and mean firing rate during the development (Fig. 2E), keeping high values of AE also after the DIV max (DIV42 for HD and DIV49 for LD). The mean bursting rate (MBR) reflected the development of the firing trend, showing maximum values at DIV42 and DIV49 for high- and low-density cultures, respectively. Moreover, the MBR profile of the high-density networks highlighted a more unstable trend with respect to the progressive increase of the low-density ones (Fig. 2F). Nevertheless, the percentage of RS constantly decreased over time, approaching values close to zero, depicting a progressively more stable and organized network (Fig. 2G). Finally, the NBR profile reflected the MBR activity with a shift in the insurgence and maximum value of the low-density cultures with respect to the high-density ones (Fig. 2H).

### Differential effects of culture media on both neuronal network and single cell maturation at varying cell densities

To evaluate differences between Neurobasal and BrainPhys media, we compared the electrophysiological properties of cultures seeded at identical cell densities both at the neuronal network level and the single-neuron level. We first analyzed high-density networks (supplementary Fig. 2A–D), followed by low-density networks (supplementary Fig. 2E–H). Analysis of MFR and network-related parameters (i.e., MBR, the percentage of RS, and NBR) clearly indicated that BP, under HD conditions, promoted accelerated network maturation, followed by a rapid functional decline (beginning at DIV 28). In contrast, NB did not induce early maturation to the same extent as BP, but supported a more gradual development, leading to higher and more sustained firing activity from DIV 35. Under LD conditions, both MFR and MBR showed a progressive maturation with comparable dynamics between NB and BP. Nevertheless, even in this context, NB supported network activity over a longer time window compared to BP.

**Fig. 3:**
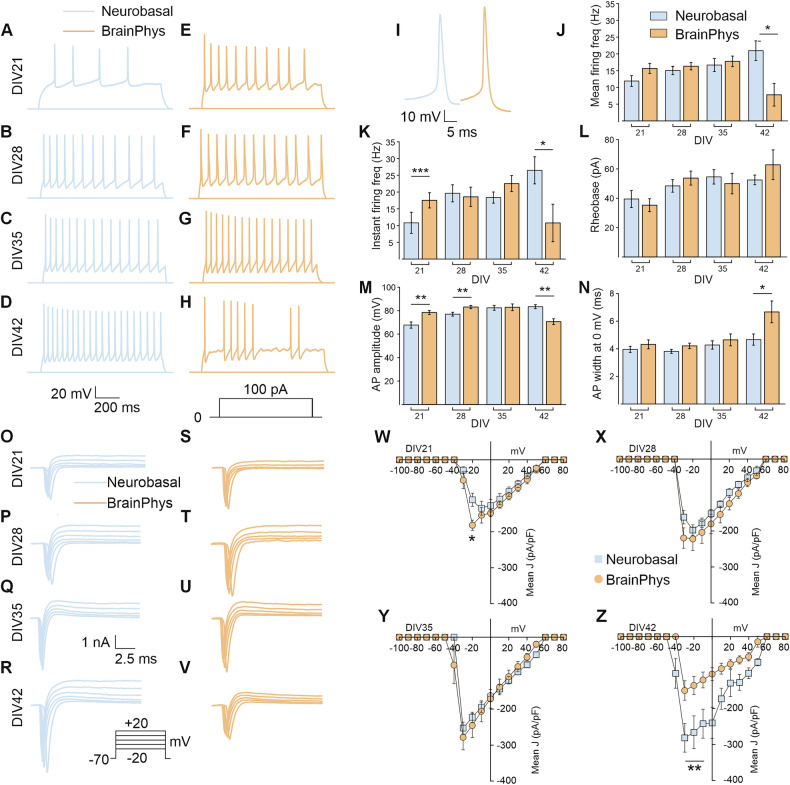
Action potential and voltage-gated sodium currents activity of iGluNeurons grown in Neurobasal and BrainPhys culture media. Whole cell current clamp recording of the action potential (AP) firing activity elicited after the injection of 100 pA depolarizing current and recorded from iGluNeurons cultured in either Neurobasal (blue) or BrainPhys (red) at four timepoints of in vitro differentiation: DIV21 (**A**, **E**), DIV28 (**B**, **F**), DIV35 (**C**, **G**), DIV42 (**D**, **H**). **I** Comparison between single action potential spikes recorded from NB- (light blue) and BP- (orange) cultured iGluNeurons. **J**–**N** Quantification of the main AP parameters assessed at each time point of in vitro differentiation in both experimental conditions (NB or BP). Mean firing frequency (Hz, **J**), Instantaneous firing frequency (Hz, **K**), Rheobase (pA, **L**), AP amplitude (mV, **M**), action potential width (ms, **N**). Representative whole-cell current families recorded from iGluNeurons cultured in NB (blue) or BP (red) at each timepoint of differentiation: DIV21 (**O**–**S**), DIV28 (**P**–**T**), DIV35 (**Q**–**U**), DIV42 (**R**, **V**). **W**–**Z** Mean I/V relationship of inward current densities J (pA/pF) recorded at each timepoint in both experimental conditions. Data are expressed as means ± SEM. *p < 0.05; **p < 0.01; *** to *p* < 0.001. The final dataset consists of *n* = 24–37 iGluNeurons per group, derived from at least three independent experiments across three distinct iPSC lines.

To assess how culture media influence single-cell electrophysiological activity, we performed whole-cell patch-clamp recordings in both current-clamp and voltage-clamp configurations at four DIVs (DIV 21, 28, 35, and 42) corresponding to different stages of in vitro differentiation. At each time point, iGluNeurons cultured in either NB or BP were analyzed. We first investigated whether NB and BP affected the action potential (AP) firing properties of iGluNeurons. In the current-clamp configuration, AP firing was elicited by linearly increasing depolarizing current injections (Fig. 3A–H). A 100-pA depolarizing current injection reliably evoked AP firing, with firing frequency progressively increasing over time [1]. Interestingly, although a significant increase in the number of the elicited APs was found at DIV 21 in iGluNeurons cultured in BP compared to the NB (Fig. 3A, E), no remarkable differences were observed in both conditions at DIV 28 (Fig. 3B, F) and 35 (Fig. 3C,G). Notably, a marked decline in the number of evoked APs was observed in iGluNeurons cultured in BP, but not in NB, at DIV 42 (Fig. 3D–H). Significant differences in mean firing frequency between iGluNeurons cultured in NB and BP media were observed only at DIV42 (Fig. 3J). However, instantaneous firing frequency was significantly higher at DIV 21 and lower at DIV 42 in BP-cultured neurons compared to those in NB (Fig. 3K). No significant changes were detected in the rheobase under either condition (Fig. 3L). When analyzing single AP (Fig. 3I), we observed that AP amplitude in BP-cultured iGluNeurons was higher at DIV 21 and 28, but lower at DIV 42, compared to NB (Fig. 3M). Finally, AP width was significantly greater in BP-cultured cells than in NB at DIV 42 (Fig. 3N). Then, we have also studied the spontaneous firing activity under both conditions. Current-clamp recordings were performed at each differentiation time point by applying a constant depolarizing current to bring the membrane potential to the theoretical AP-threshold of −40 mV (Supplementary Fig. 3A–I). No significant differences were observed between the conditions, except at DIV 42. Because changes in AP amplitude are typically linked to alterations in voltage-gated Na^+^ channel function, we performed voltage-clamp recordings of macroscopic currents to further investigate this aspect [2]. Inward currents elicited by the application of depolarizing steps from −100 to + 80 mV (50-ms duration, *V*_holding_ of −70 mV) were recorded at each time point of in vitro differentiation (Fig. 3O–V). These depolarizing steps elicited fast-inactivating inward currents, consistent with voltage-gated Na^+^ currents, which showed a slight increase in current density (pA/pF) amplitude in BP-cultured neurons at DIV 21. Conversely, at DIV 42, the current density amplitude was markedly reduced in BP- compared to NB-cultured iGluNeurons (Fig. 3W–Z). No notable differences were observed between the two culture conditions at DIV 28 and 35. All these results indicate that, based on patch-clamp analysis under well-defined culture conditions, NB medium supported gradual and sustained iGluNeuron maturation over time, while BP promoted earlier maturation, but its effect declined more rapidly.

Based on patch-clamp results, we further investigated the morphofunctional properties of our neuronal cultures exposed to NB and BP media over time. We focused our analysis on cultures maintained in NB or BP at DIV 21 and DIV 42, using neuronal markers including MAP2 (neuronal cytoskeleton), NeuN (post-mitotic neurons), ANKG (axon initial segment marker), and SYN1/2 (synaptic marker) to evaluate the differential contribution of each medium to neuronal differentiation and maturation.

**Fig. 4:**
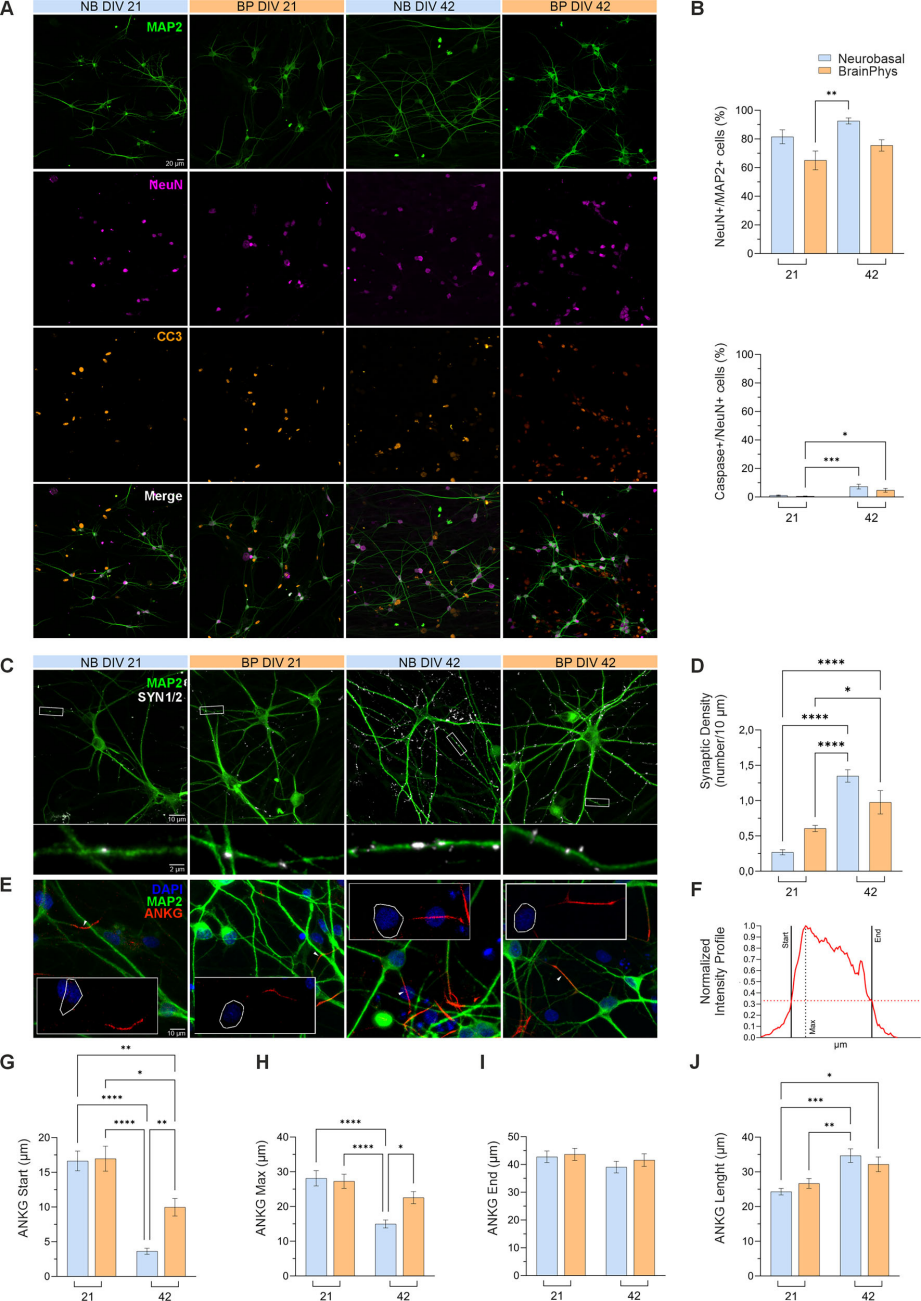
Morphofunctional characterization of iGluNeurons in Neurobasal and BrainPhys conditions. **A** Representative immunostaining of iGluNeurons marked with MAP2, NeuN and Caspase (scale bar 10 µM). **B** NeuN + /MAP2+ cells count (upper) and Caspase + /NeuN+ cells count (below). **C** Representative immunostaining of iGluNeurons marked SYN1/2 respectively (scale bar 10 µM). **D** Synaptic density (number/10 µm) quantification (mean SEM). **E** Representative immunostaining of iGluNeurons marked with ANKG (scale bar 10 µM). **F** Normalized intensity profile of ANKG marker. **G**–**L** ANKG Start, Max, End, Length quantification. Data are expressed as means ± SEM. All data represented were obtained from at least three independent preparations across three different iPSC line *n* = 23–27 images; 3 coverslips/preparation**/** iPSC line.

Quantitative analyzes were performed by counting NeuN⁺/MAP2⁺ and Caspase⁺/NeuN⁺ cells using ImageJ, while ANKG and SYN1/2 signals were analyzed by imaging-based morphometric approaches (see “Material and methods”). Neuronal maturation was assessed by quantifying the proportion of NeuN⁺/MAP2⁺ cells at matched DIV. A non-significantly trend toward a higher number of mature neurons was observed in cultures maintained in NB compared to BP at equivalent DIV (Fig. 4A, B upper). To evaluate neuronal survival over time, apoptosis in mature neurons was quantified as the percentage of Cleaved Caspase-3⁺/NeuN⁺ cells. A significant increase in apoptotic mature neurons was detected between DIV21 and DIV42, independently of the culture medium used. Despite this temporal increase, the overall proportion of apoptotic NeuN⁺ neurons remained low, consistently around 5%, even at later time points (Fig. 4A, B lower).

We next examined two key neuronal compartments essential for functional maturation—the synapse and the axon initial segment (AIS)—by immunostaining for SYN1/2 and ANKG, respectively (Fig. 4C, E). With respect to synaptic density assessed by SYN1/2 immunoreactivity, we observed an increase between DIV 21 and DIV 42 in both media, that was more pronounced in NB compared to BP cultures (Fig. 4C, D). For AIS analysis, ANKG signal start (distance from the soma), maximum intensity (peak), end (distance from the soma), and total length were measured, as schematized in Fig. 4F. Between DIV 21 and DIV 42, we observed a trend toward AIS relocation closer to the soma (Fig. 4G) and a reduction in AIS maximum intensity (Fig. 4H), both features associated with increased neuronal maturation [57]. Although AIS parameters were comparable between media at DIV 21, NB cultures exhibited significant differences between DIV 21 and DIV 42, whereas BP cultures did not, indicating more pronounced maturation in NB conditions (Fig. 4G–J). Overall, these findings indicate that NB medium supports a higher proportion of synapses and mature AIS structures compared to BP under the conditions tested. Taken together, the results from immunocytochemistry, MEA and patch-clamp functional characterization confirmed a significant contribution of BrainPhys medium to the acceleration of neuronal maturation. This effect was particularly pronounced in high-density cultures, although it was also evident under low-density conditions. In contrast, Neurobasal supported a more gradual developmental trajectory: cultures reached their peak of firing activity and functional maturation of the compartment later than their respective BrainPhys counterparts, but were able to reach a higher level of neuronal activity for a longer time.

### Electrophysiological signatures of the four culture conditions with the highest spontaneous firing

**Fig. 5:**
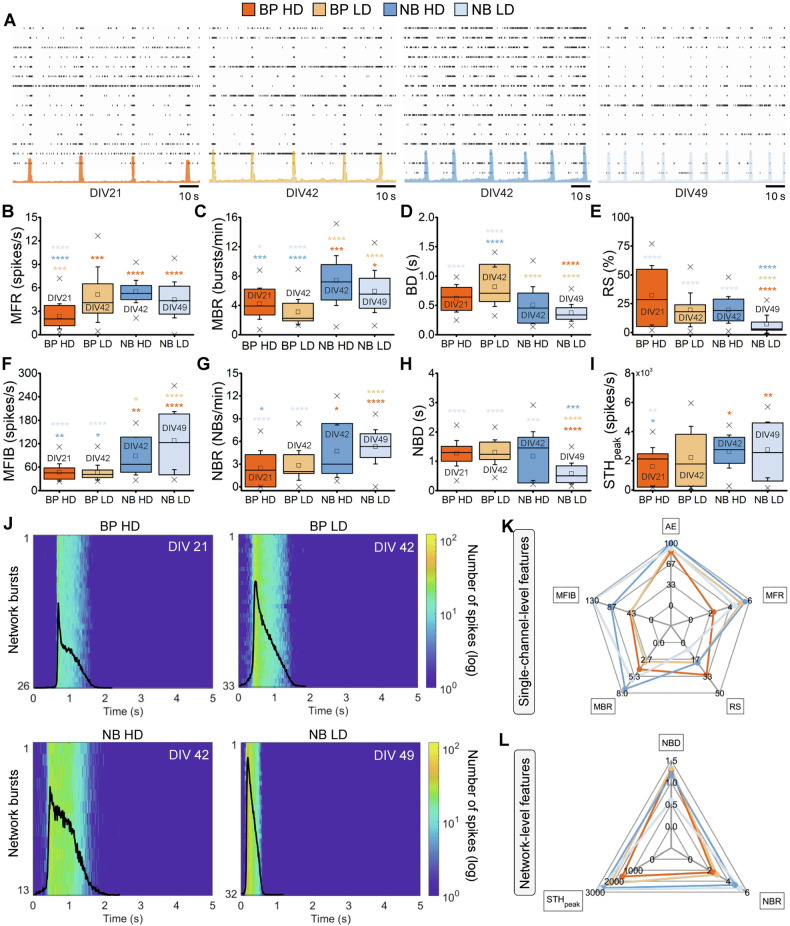
Electrophysiological comparison of the four different configurations at their maximum maturation. **A** Rapresentative raster plots of each max condition of the four different configurations at their maximum maturation. Box plots for each condition of: **B** Mean Firing Rate (MFR), **C** Mean Bursting Rate (MBR), **D** Burst Duration (BD), **E** Random Spikes (RS), **F** Mean Frequency Intra Burst (MFIB), **G** Network Bursting Rate (NBR), **H** Network Burst Duration (NBD), and **I** amplitude of the spike time histogram (STH_peak_). Data are shown as the mean (line), median (square), standard deviation (whiskers), and minimum and maximum values (crosses). *p < 0.05, ** to *p* < 0.01, ***p < 0.001, and ****p < 0.0001. **J** Aligned network bursts with respect to the peak of the longest network burst for a representative neuronal culture for each condition at the DIV max. **K** Radar plot of the single-channel-level related features for each condition. **L** Radar plot of the network-level related features for each condition. Data are shown as means ± SEM. Data were obtained as described in the MEA recordings and data analysis section. All *p*-values of the computed statistical comparisons, including the significant ones, are reported in the Supplementary Table 1B.

To evaluate the combinatorial effect of medium and cell density on the development and maturation of the neuronal networks (representative raster plots Fig. 5A), we compared the electrophysiological features of each configuration at their DIV of maximum activity, i.e., the ones reported in Fig. 1D. Concerning the mean firing rate (Fig. 5B), the BP-HD configuration showed significant differences with respect to the other ones, highlighting a lower firing activity at the peak of development. Indeed, despite this configuration promoting an accelerated maturation with the highest activity level already reached by DIV 21, this early maturation was not sustained, as shown by the following decline in all parameters starting from DIV 28 (Fig. 2A–D). Regarding the bursting pattern, the neuronal network in NB medium (NB_net_) exhibited a higher frequency of bursting events (Fig. 5C) and revealed the NB-LD configuration as the most organized network among all the configurations, with the lowest burst duration (Fig. 5D) and random spiking activity (Fig. 5E). On the other hand, the neuronal network in BP medium (BP_net_) displayed longer shapes (Fig. 5D) and a reduced mean frequency intra-burst (Fig. 5F) with respect to NB_net_. Notably, the BP-HD configuration proved to be the least organized network among all the configurations, showing the highest percentage of random spiking activity at the peak of the development (Fig. 5E). Those aspects were also reflected in the network bursting pattern. Indeed, the NBR showed lower values in the neuronal networks cultured in BP (Fig. 5G), which were also characterized by longer network events (Fig. 5H) with a lower amplitude (Fig. 5I, J). In this scenario, the main differences were related and highlighted by the employment of different culture media rather than different densities. Indeed, few statistical differences arose between the high- and low-density configurations of the networks cultured with the same medium (i.e., BP-HD vs BP-LD and NB-HD vs NB-LD). This aspect was further confirmed by considering all at once the single-channel-level and the network-level related features. Thus, by evaluating the radar plots of such parameters (Fig. 5K, L), the neuronal dynamics exhibited by the various cultures demonstrated comparable distributions of the parameters when comparing low- and high-density NB_net_ and low- and high-density BP_net_. On the other hand, a clear distinction can be made between the NB_net_ and BP_net_ distributions. In conclusion, both aspects related to single-channel level features, i.e., firing and bursting pattern (Fig. 5K), and to the network-level (Fig. 5L) highlighted differences among the configurations in the maximum of the development of NB_net_ and BP_net_, allowing them to be clearly distinguished from each other by different characteristics.

### iGluNeuron proteomics: neuronal differentiation-associated proteins and culture condition-specific signatures

In most published studies on iGluNeuron, protein expression is inferred from either bulk or single-cell gene expression data. While these approaches are highly informative, they represent indirect assessments of protein expression. In iGluNeurons culture system, the main limitation to applying proteomic analyzes lies in the co-culture system involving two different species, which presents significant challenges for accurate taxonomic assignment. To overcome this issue, we propose an algorithm that robustly filters Homo sapiens-specific peptides, thereby limiting the interference of rat astrocyte peptides in downstream association tests (see “Materials and methods”).

**Fig. 6:**
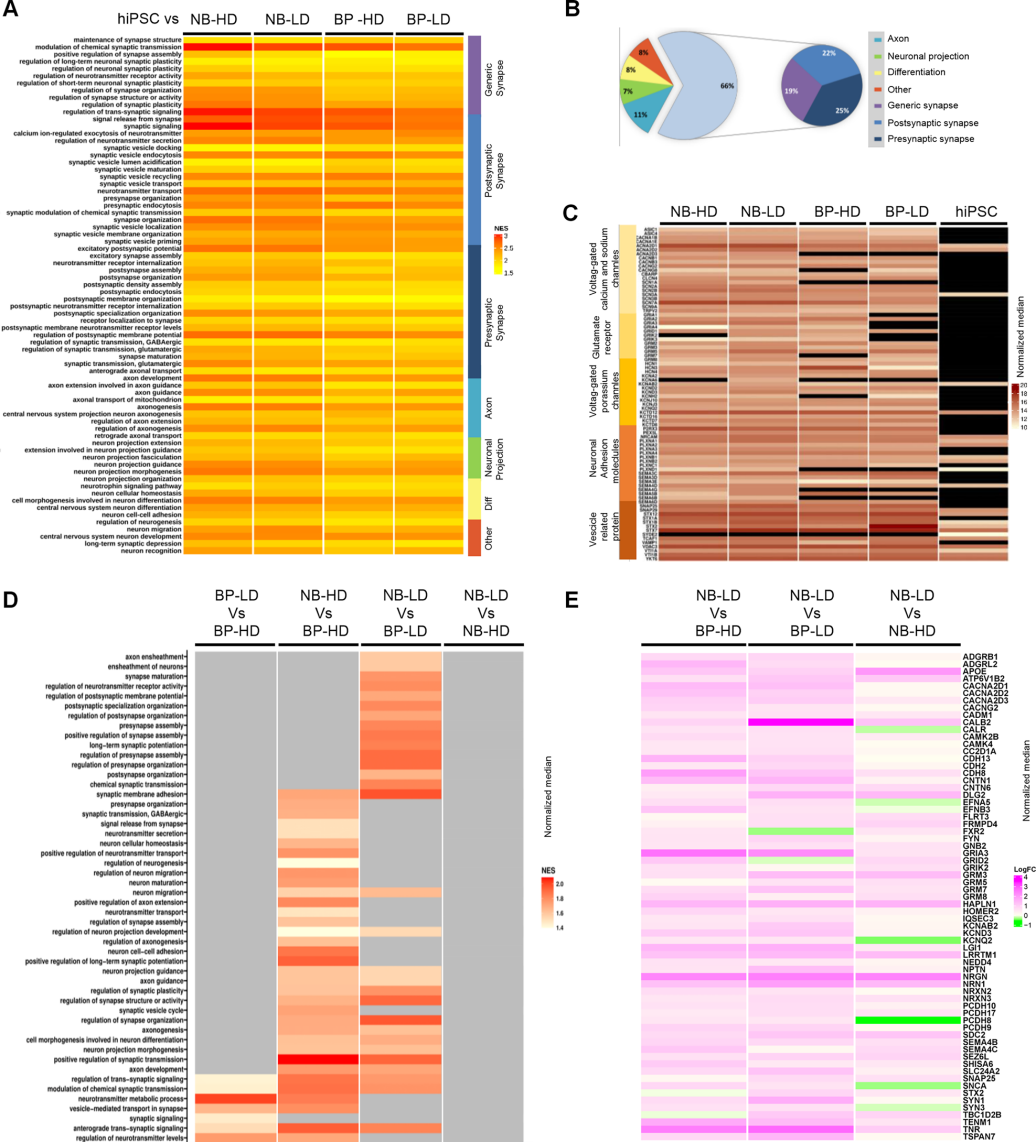
Proteome comparison of the four different configurations at their maximum development. **A** Comparison iGluNeurons to hiPSC. Heatmap representation of the significant “Gene Ontology- Biological Process” neuronal-related ontologies inferred by gene set enrichment analysis common to all contrasts between iGluNeuron conditions versus hiPSC depicted by their normalized enriched scores. Across all 4 contrasts, each gene set associates with positive log2 fold change trends of the corresponding enriching proteins. **B** Pie chart illustrating the categorization of enriched pathways into major groups. **C** Heatmaps showing median abundance profiles of a protein set comprising 82 informative proteins, compared across iGluNeuron culture conditions and the hiPSC phenotype (black: not detected value). **D** Heatmap representation of the significant “Gene Ontology- Biological Process” neuronal-related ontologies inferred by gene set enrichment analysis across all contrasts defined between iGluNeuron conditions depicted by their normalized enriched scores (gray: non-significantly enriched ontology). **E** Heatmaps showing the comparison of NB LD versus all the other conditions expressed as log fold change (white: not detected). Data were obtained as described in the proteomics data analysis section.

First, we compared the proteomes of the four conditions previously identified as exhibiting the highest spontaneous firing activity (BP-HD, BP-LD, NB-HD, NB-LD) with those of their respective hiPSCs. We focused our analysis on neuron-specific enriched pathways. As shown in Fig. 6A, a core set of neuron-specific pathways was consistently upregulated relative to hiPSCs—regardless of the experimental condition—reflecting NGN2-driven differentiation. The top upregulated enriched pathways were primarily associated with pre- and post-synaptic compartments, axons, and neuronal projections, as illustrated in the pie chart in Fig. 6B. Around 2500 proteins are differentially expressed compared to hiPSCs and were shared across all four conditions (Supplementary Table 2). Supplementary Fig. 4A presents the most downregulated pathways, which largely correspond to processes related to proliferation and differentiation, such as DNA-templated DNA replication and rRNA processing [58]. Finally, to assess the expression of specific proteins known to play key roles in neuronal function, we interrogated the HUGO Gene Nomenclature Committee (HGNC) gene lists for voltage-gated ion channels, glutamate receptors, synaptic proteins, vesicle-related proteins, and SNARE components. Additionally, we defined a new category, “Neuronal Adhesion Molecules,” which includes plexins, NRCAM, NCAM, and semaphorins. The proteins belonging to these categories were highly represented and consistently upregulated under all conditions (Fig. 6C). Furthermore, we used the OMIM website (https://omim.org/) to associate each upregulated neuron-specific protein (listed in Supplementary Table 2) identified in the comparison with hiPSCs—with a corresponding neurological disorder. The range of diseases that can potentially be modeled in vitro using this differentiation system spans from numerous monogenic to polygenic conditions, encompassing both neurodevelopmental and neurodegenerative disorders (Supplementary Table 3).

Second, we focused on pathways that were differentially enriched and regulated across the four conditions to analyze the contribution of medium and cell density. Notably, differentially expressed pathways were either absent (in the comparison between NB-LD and NB-HD) or minimal (in the comparison between BP-HD and BP-LD) when conditions shared the same culture medium. In contrast, when comparing culture settings with matched cell density but different media, striking differences emerged—consistently favoring the upregulation of enriched pathways related to synapse in the NB-containing conditions (Fig. 6D). Indeed, the significantly enriched differentially expressed pathways are predominantly related to the organization and activity of the synaptic compartment, such as regulation of presynaptic organization, regulation of postsynaptic organization, regulation of synaptic plasticity, synaptic membrane adhesion, synaptic vesicle cycle, regulation of synaptic activity (see Supplementary Table 4 for details on the proteins associated with each pathway). Finally, we interrogated the SynGO gene lists to identify proteins differentially expressed across conditions compared to the NB-LD condition. As shown in Fig. 6E, the proteins upregulated in the NB-LD cultures were predominantly associated with the glutamatergic synapse. Specifically, we observed an increased expression of synaptic proteins involved in presynaptic calcium channel function (CACNA2D1, CACNA2D2, CACNA2D3), neurotransmitter release (SYN1, SYN3, ATP6V1B2), synaptic structural organization (ADGRL2, NRN1, TSPAN7, TNR, TNEM1, LGI1 and LRRTM1) and synaptic adhesion molecules (CDH13, CDH2, CDH8, CNTN1, CNTN6, PCDH10, PCDH17, PCDH8, PCDH9). In addition, several glutamatergic postsynaptic receptors, both ionotropic and metabotropic, were upregulated, including GRIA3, GRM3, GRM7, together with key regulators of the glutamatergic postsynapse (SHISA6, FRMPD4, CACNG2, CAMK2B, DLG2, HOMER2, NRGN).

Overall, these results indicate that the proteomic profile is primarily driven by culture medium composition rather than plating density. Consistently, as shown in Fig. 6D, differentially enriched pathways emerge mainly when comparing different media at matched cell density. Furthermore, comparison of the NB-LD condition with the other culture conditions at the protein level revealed higher expression of proteins related to glutamate receptors and their regulators, synaptic structural components, synaptic adhesion molecules, and neurotransmitter release machinery.

### Functional synaptic characterization of the best culture conditions to maintain long term and highly functional network

**Fig. 7:**
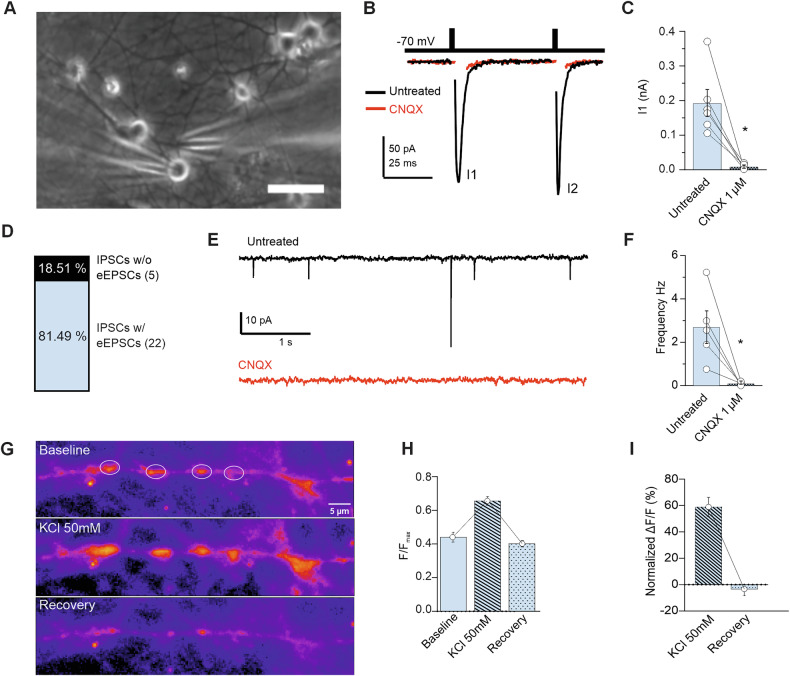
iPSC-derived neurons exhibit excitatory synaptic currents. **A** Phase-contrast micrographs of the experimental setup showing the stimulation electrode positioned near the recorded neuron (right) and the patch-clamp recording electrode patching an iGluNeurons. Scale bar: 100 μm. **B** Representative evoked excitatory postsynaptic currents (eEPSCs) recorded from low-density plated iGluNeurons, elicited by clamping the cell at −70 mV and applying paired minimal stimulation voltage pulses with an inter-stimulus interval of 50 ms. Paired-pulse stimulation was delivered every 10 s. In all graphed currents, stimulation artifacts were blanked for clarity. eEPSCs recorded in neurons perfused with control standard extracellular solution and after acute application of the AMPA receptor antagonist CNQX (10 μM; red). **C** Bar-graph showing the mean (±SEM) eEPSC amplitude measured in iGluNeurons under control conditions and following CNQX (10 μM) treatment, as in **B**. **D** The graph shows the percentage of responsive (light blue, 81.49%) and non-responsive (black, 18.51%) iGluNeurons studied as in **B**. Superimposed symbols represent mean eEPSC values from individual cells recorded before and after CNQX application. **E** Representative traces of spontaneous EPSCs (sEPSCs) recorded from low-density iGluNeurons in control standard extracellular solution and after acute CNQX treatment, as in **B**. **F** Bar-graph of the mean (±SEM) sEPSC frequency recorded in iGluNeurons under control conditions and following CNQX (10 μM) acute perfusion, as in **E**. Data are expressed as means ± SEM from the indicated numbers of cells recorded from at least three independent cell culture preparations. *p < 0.05, paired Wilcoxon test. **G** Representative images of iGluNeurons infected at DIV35 with the synaptic reporter SypHy and imaged at DIV50 during the baseline condition (top), the response to 50 mM KCl stimulation (middle), and the subsequent recovery phase (bottom); in the baseline panel, a representative set of ROIs used for analysis is indicated. **H** Fluorescence responses are quantified as F/Fmax. **I** The percentage change in fluorescence (Δ*F*/*F*%) is calculated for KCL and recovery relative to baseline. Data were obtained as described in the Patch-clamp recordings and data analysis and SynaptopHluorin imaging.

Based on the data obtained along this study, we identified that the culture conditions that best support iGluNeurons in terms of electrophysiological properties at both the single-cell and network levels, proteomic profile, and the organization of key neuronal subcellular compartments essential for neuronal activity, consist of a Neurobasal-based medium combined with a plating density of 1200 cells/mm^2^. We next investigated whether iGluNeurons possess a functional synaptic machinery capable of supporting synaptic vesicle release. To address this, we performed whole-cell patch-clamp recordings of evoked excitatory postsynaptic currents (eEPSCs) elicited by extracellular stimulation at the single-cell level (Fig. 7A) and live imaging with SypHy probe (Fig. 7G). eEPSCs were recorded from low-density plated iGluNeurons using paired-pulse stimulation, which produced currents with rapid kinetics characteristic of excitatory synaptic transmission and were completely blocked by the AMPA receptor antagonist CNQX (Fig. 7B, C) [59]. This presynaptic stimulation protocol allowed us to examine short-term synaptic plasticity by comparing the first (I1) and second (I2) evoked responses. Under these conditions, the I2 response was transiently enhanced relative to I1, indicating paired-pulse facilitation typical of excitatory synapses [60]. Overall, approximately 90% of iGluNeurons exhibited detectable synaptic currents (Fig. 7D). Recordings of spontaneous network activity showed that these synaptic properties, including CNQX sensitivity, were also preserved at the network level (Fig. 7E, F). A quantitative analysis of synaptic properties at both the single-cell and network levels is summarized in Supplementary Table 4. SypHy imaging, performed in low density plated iGluNeurons, revealed a significant increase of fluorescence signals along neurites upon KCl stimulation and recovery to the baseline at the end of the stimulation (Fig. 7H, I)

## Discussion

Numerous studies have demonstrated that NGN2-mediated differentiation enables the rapid conversion of hiPSCs into functionally mature excitatory neurons within a remarkably short timeframe [1, 6, 54]. Through a systematic review of the literature on NGN2-mediated differentiation toward iNeurons, we identified a significant lack of protocol standardization across research groups. The few attempts to compare key conditions influencing culture development and maturation have typically addressed only one variable at a time, and often assessed only a limited set of parameters [17, 41, 54].

We here compared all combinations of three critical variables, resulting in a matrix of eight distinct conditions. This design allowed us to evaluate the combined contribution of medium, coating, and cell density across a wide range of functional parameters, including single-cell and network-level electrophysiological properties, as well as total protein expression. Initial assessments using MEAs showed that substrate coating with PEI or PLO had no significant effect on the network electrophysiological parameters. In contrast, both culture medium and cell density clearly influenced single-cell and network electrophysiological properties.

While all the analyzed conditions successfully led to the development of functionally active networks characterized by typical patterns of dynamics found in vitro, such as firing, bursting, and network bursting activity, different timing and characteristics were observed changing the culture media and cell densities.

Considering the variables of medium and cell density separately, we found that BrainPhys, compared to Neurobasal, promotes earlier network maturation — as previously observed [61, 62]— but followed by a more rapid decline once peak activity is reached. While the specific component(s) of BrainPhys responsible for this accelerated maturation remain unclear, a previous study has suggested that its low glucose concentration (2.5 mM in BP vs. 25 mM in NB) may contribute to the subsequent drop in the network activity [54]. From the perspective of network dynamics, cultures maintained in BrainPhys exhibited reduced overall firing, bursting, and network bursting frequency and were characterized by longer bursting and network bursting events with lower amplitudes. Immunocytochemical analyzes indicate that most neurons are already postmitotic by 21 DIV, independently of the culture medium. In terms of cell viability, only a modest increase in apoptosis was observed over time, reaching approximately 5% at DIV42. Notably, although neurons are postmitotic at early time points, they do not yet exhibit full morphofunctional maturity. Consistent with this observation, we detected a progressive increase in synaptic density and AIS remodeling over time in both culture conditions, with these changes being more pronounced in Neurobasal cultures. Indeed, an increase in synaptic contacts and a closer positioning of the AIS to the soma have been correlated with neuronal maturation. Synapsin-1/2 was selected as a robust pre-synaptic marker to reliably quantify synaptic density. While glutamatergic-specific markers such as VGLUT1/2 could, in principle, provide additional information, the available antibodies showed lower staining efficiency and signal robustness under our experimental conditions. Nevertheless, the glutamatergic identity of the synaptic activity was independently demonstrated by both proteomic profiling and functional synaptic analyzes. These maturation markers align with functional synapse formation and electrophysiological maturity observed. Together, these findings suggest that Neurobasal medium more effectively supports the synapse development and the establishment of a mature AIS compared to BrainPhys.

Increasing cell density also accelerates the timing of network maturation, but this effect is short-lived when compared to low-density cultures. In contrast, a lower density supported a more gradual maturation trajectory, ultimately reaching higher levels of network complexity and maintaining them over longer periods in culture.

When considering medium and cell density in combination, BrainPhys and high-density conditions (BP-HD) displayed a synergistic effect, with maximal network maturation at DIV 21, followed by a rapid decline by DIV 28. Thus, the use of BrainPhys as a medium and high density can be a winning combination if used in studies investigating a single time point in the peak of the activity over short times; on the contrary, it is not the most suitable for developmental studies, which require the maintenance of networks in long-term cultures. Conversely, cultures in Neurobasal at high density (NB-HD) show a maturation profile resembling that of low-density Neurobasal (NB-LD) cultures, although shifted earlier by approximately 1–2 weeks and with increased variability in the functional parameters. As a result, these conditions may be preferable for the assessment of maturation and development of networks over time, with the disadvantage of taking longer to exhibit sustained and synchronized activity (about 35 DIV). Notably, both NB-LD and BP-LD conditions support a similar progressive developmental trajectory, ultimately achieving greater and more sustained network maturity compared to their high-density counterparts.

Finally, we further characterized iGluNeuron differentiation through proteomic profiling, addressing a key limitation of previous studies that relied primarily on transcriptomic data to infer protein expression [15–17]. By developing and applying a robust taxonomic filtering algorithm, we ensured the accurate quantification of human-specific proteins in the human–rat co-culture system. This approach provides a direct and unbiased, yet deliberately conservative, proteomic profiling, as it restricts the analysis to proteins unambiguously assigned to the human proteome, potentially excluding proteins shared between human and rat. Analysis of the four conditions exhibiting the highest spontaneous firing activity (BP-HD, BP-LD, NB-HD, NB-LD) revealed a consistent upregulation of neuron-specific pathways—primarily related to synaptic and axonal compartments—across all iGluNeuron configurations compared to hiPSCs, regardless of the culture conditions. These findings reflect a conserved NGN2-driven differentiation program at the proteomic level. Furthermore, we demonstrated that differences in culture medium composition, rather than cell density, are the primary drivers of proteomic variability among iGluNeurons. Neurobasal-based media consistently supported a higher enrichment of pathways involved in the regulation of presynaptic and postsynaptic organization, regulation of synaptic plasticity, synaptic membrane adhesion, synaptic vesicle cycle and synaptic activity (Fig. 6D). Taken together, data obtained from immunocytochemistry and electrophysiological recordings at both the single-cell and network levels clearly demonstrate that the NB-LD condition enables neuronal cultures to reach the highest levels of functional activity and to sustain them over time. This convergence of structural and functional evidence indicates that NB-LD strongly promotes synaptic functional maturity. Indeed, comparisons between NB-LD and the other culture conditions allow the identification of specific molecular features that may account for its distinctive properties. In particular, (i) the increased expression of presynaptic proteins such as CACNA2D1/2/3 [63, 64] supports enhanced excitatory synaptic neurotransmission, potentially leading to greater synaptic efficacy, neuronal excitability, and synapse formation. Likewise, the upregulation of ATP6V1B2, SYN1, and SYN3—key components of the neurotransmitter release machinery—reflects an advanced stage of synaptic maturation and is consistent with the higher levels of network activity observed [65–67]. (ii) The enrichment of adhesion molecules and extracellular matrix proteins suggests improved synaptic organization and stabilization, which are essential for the maintenance of mature synaptic contacts over time. (iii) In addition, the specific increase in GRIA3 [68], together with the expression of GRIA1 and GRIA2, points toward an enhancement of fast excitatory synaptic transmission, a hallmark of functionally mature glutamatergic synapses. (iv) Finally, the differential regulation of multiple regulatory and scaffolding proteins associated with glutamatergic synapses further supports the notion that NB-LD fosters a synaptic environment conducive to functional refinement and sustained network-level activity together with key regulators of the glutamatergic postsynapse (SHISA6, FRMPD4, CACNG2, CAMK2B, DLG2, HOMER2, NRGN) [69, 70]. These findings enable the assessment of the presence of specific target proteins relevant to disease modeling and drug screening. Finally, synaptic functionality was characterized by patch-clamp recordings and SypHy assay, which confirmed the presence of active synapses, the primary role of AMPA receptors and their associated regulatory proteins in driving iGluNeuron activity, as evidenced by the marked suppression of synaptic responses following CNQX administration. The comprehensive electrophysiological, morphofunctional and proteomic profiling of iGluNeurons across diverse culture conditions offers a powerful framework for future research, establishing a strategic reference point for both the evaluation of target protein expression and the rational selection of functional assays in disease modeling and drug screening.

## Materials and methods

### Generation and maintenance of hiPSCs-derived neuronal cultures

The three human induced pluripotent stem cell (hiPSC) lines used in this study were obtained from two different sources. Two hiPSC lines were provided by the Cell Line and DNA Biobank from Patients Affected by Genetic Diseases (Istituto G. Gaslini, Genoa, Italy), a member of the Telethon Network of Genetic Biobanks. The third hiPSC line was a commercial line (ASE-9211) purchased from Applied StemCell. The use of these cell lines was approved by the Ethical Committee of Regione Liguria (IRB project no. GTB18001), and their detailed characterization has been previously described [1]. Informed consents were obtained from patients. To generate iGluNeurons cultures, we followed the pipeline previously published [1]. Briefly, 300.000 hiPSCs-NGN2 were plated as single cells in pre-differentiation medium with doxycycline (4 µg/ml) and neurotrophic factors (i.e., BDNF 10 ng/ml, NT3 10 ng/ml and mouse Laminin 1 µg/mL) for 3 days. After 3 days, neuroprogenitors (NPCs) were frozen using the iNeurons freezing solution (80% KnockOut Serum Replacement – Gibco – and 20% DMSO - Sigma) and preserved until the experiments’ days. For the realization of this study, eight distinct conditions were established based on the combinations of different cellular densities, coatings, and media (supplementary Fig. 1A, B). In particular, two cellular densities were implemented: low density (LD) at 1200 cells/mm^2^ and high density (HD) at 4800 cells/mm^2^ for the multi-electrode arrays (MEA) analysis. Due to the technical constraints of the patch-clamp technique, recordings were conducted exclusively under LD conditions. Concerning the adhesion factors, Poly-L-Ornithine hydrobromide (PLO, 50 µg/mL, Sigma-Aldrich) and Polyethylenimine (PEI, 50 µg/mL, Sigma-Aldrich) were used, while Neurobasal (Gibco) and BrainPhys (StemCell) were utilized as culture media. The day before thawing NPCs, the surfaces of MEA devices (for electrophysiological recordings) and petri dishes (for patch-clamp recordings) were coated overnight with PLO or PEI to promote cellular adhesion. The day of plating, PEI and PLO solutions were washed three times with UltraPure water (Invitrogen) and the surfaces were coated with mouse Laminin (10 µg/mL in DMEM/F12, Sigma) for 2 h at 37 °C. NPCs were thawed and co-plated with primary rat astrocytes (ratio 1:3) at the final density of 1200 (LD) or 4800 (HD) cells/mm^2^ on CytoView 48 wells (Axion BioSystems Inc), petri dishes (3 cm of diameters), or IBIDI (Twin Helix, 80806). The neuronal networks were maintained for 56 Days In Vitro (DIV). The medium was half-replaced 2 days per week with NB or BP supplemented with B27 2% (Gibco), BDNF 10 ng/mL (Thermofisher), NT3 10 ng/mL (Thermofisher), mouse Laminin 10 ng/mL (Sigma) and Doxycycline 4 μg/mL (removed from DIV14 onwards). In parallel with electrophysiological investigations, to perform proteomic analysis, iGluNeurons cultured with NB and BP were analyzed at DIV 21, DIV 42 and DIV 49.

### Systematic review methodology

We conducted a systematic search of the PubMed database up to March 2025 using the following search string: “NGN2” AND “induced neurons”. The initial search retrieved 159 records. In the first phase, titles and abstracts were screened for relevance based on predefined inclusion criteria (NGN2-driven differentiation). This led to the exclusion of 39 articles. Subsequently, we assessed the full text of the remaining 120 articles, from which 56 studies met the eligibility criteria (differentiation starting from hPSC and differentiation to glutamatergic neuron) and were included in the final review. These studies were published between 2013 and 2025.

### MEA recordings and data analysis

The neuronal networks were cultured on commercial 48-well MEAs (CytoView MEA plate, Axion BioSystems, Atlanta, GA, USA). Each well incorporates 16 electrodes with a 50 μm diameter and arranged in a 4 × 4 grid with a pitch of 350 μm. Once a week, from DIV 7 up to DIV 56, the Maestro Original (Axion BioSystems, Atlanta, GA, USA) recording system was exploited to collect the electrophysiological traces of the neuronal networks for 8 min at 12.5 kHz, after a 3-min period of acclimation at 37 °C. Data were recorded by applying a band-pass filter, which high-pass-filter and low-pass filter cut-off were set to 200 Hz and 3000 Hz, respectively. The experiments were performed by collecting the data from three different batches.

Off-line data analysis was performed utilizing the Axion Software (Axion BioSystems, Atlanta, GA, USA), SpyCode [71] and custom-made in-house codes developed in MATLAB (The Mathworks, Natick, MA, USA). The spike detection was computed with a hard-threshold detection, which threshold was set to five times the standard deviation of the baseline noise. Inactive channels with mean firing rate (MFR, i.e., the number of spikes in the unit time) lower than 0.1 spikes/s were discarded [17]. We assessed the evolution of the normalized MFR profile during the days in vitro for each condition, searching for the maximum point of the neuronal cultures’ development (DIV_max_). In particular, we normalized the MFR of each culture with respect to the maximum over all the DIVs of the same culture. Once DIV_max_ was defined for each condition, the wells that had a number of active electrodes <10 over a total of 16 (62.5%) were discarded from the entire dataset (from DIV7 to DIV56). Moreover, since PEI and PLO did not show statistical differences, we merged the dataset related to PEI and PLO adhesion factors, obtaining 4 different culture conditions (i.e., BP-HD, BP-LD, NB-HD, and NB-LD). After the above-described filtering process and merging, the final dataset included *n* = 34 cultures from BP-HD, *n* = 33 from BP-LD, *n* = 45 from NB-HD, and *n* = 85 from NB-LD, derived from at least three independent experiments across three iPSC lines. Burst detection was performed by exploiting the logarithmic inter-spike-interval distribution [72] with a minimum burst composition of 5 spikes. An active channel was considered as bursting when its MBR (i.e., the number of bursts per minute) was >0.4 bursts/min. From the burst detection, we extrapolated the random spikes, defined as the percentage of spikes not belonging to a burst, the burst duration, and the mean frequency intra-burst. Finally, network bursts were defined as those in 30% of the bursting electrodes that simultaneously emitted a burst with a maximum inter-burst event interval of 100 ms. From the network burst detection, we extrapolated the network bursting rate (i.e., the number of network events per minute) and the network burst duration. Furthermore, we aligned all the network bursts of each culture with respect to the peak of the longest network burst to obtain the spike time histogram (bin size = 10 ms [56], that represents the average network burst shape of the culture, of which we computed the amplitude of the peak.

### Patch-clamp recordings and data analysis

Whole-cell patch-clamp recordings in both current- and voltage-clamp configurations have been performed using an EPC-10 amplifier (HEKA) and pulled borosilicate pipettes with a resistance of 3–5 MΩ, when filled with intracellular solution. At the beginning of each recording, access resistance and membrane capacitance were routinely measured by using the automatic compensation system of the EPC-10 amplifier. Data were acquired with PatchMaster software (HEKA) and then analyzed off-line by FitMaster software (HEKA). For further analysis, only cells with an access resistance of <15 MΩ were selected. All recordings were performed at room temperature (22–24 °C). Electrophysiological recordings in both current- and voltage-clamp configurations have been carried out using the following extracellular (Tyrode) solution containing (in mM): 140 NaCl, 2 CaCl_2_, 1 MgCl_2_, 4 KCl, 10 Glucose, 10 HEPES (pH 7.3 with NaOH). To avoid contamination ascribable to the potential synaptic transmission, D-(-)-2-amino-5-phosphono-pentanoic acid (D-AP5, 50 µM), 6-cyano-7-nitroquinoxaline-2,3-dione (CNQX, 10 µM), CGP55845 (10 µM), and bicuculline (30 µM) were added to the external solution before every experiment to block NMDA, non-NMDA, GABA_A_, and GABA_B_ receptors, respectively. The patch pipette was filled with the following intracellular solution (in mM): 126 K-Gluconate, 4 NaCl, 1 MgSO_4_, 0.02 CaCl_2_, 0.1 BAPTA, 15 Glucose, 5 HEPES, 3 ATP, 0.1 GTP (pH 7.2 with KOH). Analysis of firing activity was performed as described in refs. [1, 73, 74]. Precisely, spontaneous firing activity was recorded at the threshold potential about −40 mV, while induced firing activity was assessed throughout the injection of a linear gradient of depolarizing current (Δ = 10 pA; duration of 500 ms). Mean and instantaneous mean firing frequency were determined at 100 pA of injected current. Mean firing frequency was assessed as the ratio between the number of action potentials (APs) evoked after the injection of 100 pA of depolarizing current and the time interval between the first and the last AP spikes. Instantaneous firing frequency was estimated as the reciprocal value of the time interval between the two first elicited AP spikes. Rheobase has been measured as the minimum amount of depolarizing current needed to evoke at least a single action potential spike. AP amplitude was assessed as the difference between peak and threshold. The Na^+^ current density (J; pA/pF) was calculated as the ratio between the peak inward current recorded in voltage-clamp configuration at each depolarizing step and the cell capacitance.

Evoked excitatory postsynaptic currents (eEPSCs) were recorded at 42–49 DIV according to previously established procedures. iGluNeurons were voltage-clamped at −70 mV. Presynaptic stimulation was delivered through a glass pipette (tip diameter ~1 μm) filled with extracellular solution and positioned in contact with a neurite close to the recorded neuron in a loose-seal configuration. Electrical stimuli consisting of current pulses of variable amplitude (10–45 μA) and duration (0.1–0.3 ms) were applied using an isolated pulse stimulator (Model 2100, A-M Systems, Carlsborg, WA), evoking postsynaptic currents with short latencies (2–4 ms). For all experiments, the stimulation intensity was adjusted to the minimal level required to reliably elicit single monosynaptic responses. The excitatory nature of the evoked postsynaptic currents was confirmed by acute bath application of the AMPA receptor antagonist CNQX (10 μM). eEPSCs were recorded by superfusing the whole-cell clamped postsynaptic neuron with an extracellular solution containing (in mM):140 NaCl, 2 CaCl_2_, 1 MgCl_2_, 4 KCl, 10 glucose, 10 HEPES (pH 7.3 with NaOH). D-AP5 (50 μM) and CGP58845 (5 μM) (Tocris, Bristol, UK) were added to the extracellular solution to block NMDA and GABAB receptors, respectively. The standard intracellular solution was (in mM): 126 K Gluconate, 4 NaCl, 1 MgSO_4_, 0.02 CaCl_2_, 0.1 BAPTA, 15 glucose, 5 HEPES, 3 ATP, 0.1 GTP (pH 7.2 with KOH). QX-314 (N-2,6-dimethylphenyl carbamoylmethyl triethylammonium bromide; 10 mM; Tocris) was added to block Na^+^ currents. To analyze the paired-pulse ratio (PPR), two brief extracellular depolarizing pulses were applied to the presynaptic neuron at time interval of 50 ms and every 10 s. For each couple of eEPSCs, the PPR was calculated as I2/I1, where I1 and I2 are the amplitudes of the eEPSCs evoked by the conditioning and test stimuli, respectively. The final dataset consists of *n* = 27 iGluNeurons derived from two independent experiments across two iPSC lines.

### SynaptopHluorin imaging

iGluNeurons were transduced with the SynaptopHluorin lentivirus (SypHy) [75] at DIV 35 and imaging was acquired at DIV 60 on a wide-field inverted epifluorescence microscope (IX81; Olympus) equipped with a 40x objective and a CoolLED pE-300ultra illumination system. Images were captured at a depth of 12-bits using an Orca-ER IEEE1394 CCD camera (Hamamatsu Photonics) with 2×2 binning. Fifteen images were acquired in basal solution (Tyrode-140 mM NaCl, 2.5 mM KCl, 10 mM HEPES pH 7.4, 10 mM glucose, 2 mM CaCl_2_, 1 mM MgCl_2_, pH 7.4-baseline), followed by 15 images during KCl 50 mM stimulation and 15 images during recovery in basal solution and 15 images in 50 mM NH4Cl to obtain maximal fluorescence (Fmax). For the KCl stimulation, we used a modified basal solution with 50 mM NaCl replaced by 50 mM KCl. For the NH_4_Cl challenge, the same solution was prepared by substituting 50 mM NaCl with 50 mM NH_4_Cl. The final dataset was obtained from two different cell lines and two independent experiments.

### Immunocytochemistry

Cells were fixed for 10 min with 4% (v/v) paraformaldehyde (PFA) and 4% (w/v) sucrose, then cells were washed 3 times with Phosphate Buffered Saline (PBS) and permeabilized with 0.2% Triton X-100 in PBS for 10 min, followed by three washes with PBS. After permeabilization, samples were incubated in blocking buffer (10% Fetal Bovine Serum; FBS in PBS) for 45 min. Subsequently, primary antibodies were diluted in the incubation solution (5% FBS in PBS) and added for overnight incubation. The following antibodies were used: anti-Ankyrin G (Santa Cruz; sc-28561; 1:200), anti-MAP2 (Sigma; M9942; 1:200 and Synaptic Systems; 188003; 1:200), anti-Synapsin 1/2 (Synaptic Systems; 106004; 1:100), anti-NeuN (Millipore; MAB377; 1:200) and Caspase (Cell Signaling, #9661; 1:200).

The next day cells were washed three times with PBS for 5 min, followed by the addition of the secondary antibody Goat anti-Mouse IgG, IgM (H+L) Secondary Antibody, Alexa Fluor™ 488 (Invitrogen; Catalog # A-10680); Goat anti-Rabbit IgG (H+L) Cross-Adsorbed Secondary Antibody, Alexa Fluor™ 488 (Invitrogen; Catalog # A-11008); Goat anti-Mouse IgG (H+L) Cross-Adsorbed Secondary Antibody, Alexa Fluor™ 568 (Invitrogen; Catalog # A-11004; Goat anti-Rabbit IgG (H+L) Cross-Adsorbed Secondary Antibody, Alexa Fluor™ 594 (Invitrogen; Catalog # A-11012), Donkey anti-Goat IgG (H+L) Cross-Adsorbed Secondary Antibody, Alexa Fluor™ 488 (Invitrogen; Catalog # A-11055 (1:500) in the same dilution buffer for 1 h in the dark. The secondary antibody was then removed, and Hoechst (1:300) was added for 15 min, followed by three washes with PBS for 5 min.

### Quantitative image analysis with ImageJ

To quantify immunofluorescence intensity and count positive cells, we performed threshold analysis in ImageJ. Briefly, images were converted to 8-bit, subjected to “Auto Threshold,” and particles sized 30–200 pixels were selected. We then used “Image Calculator” to quantify double-positive cells (MAP2+/NeuN+ and NeuN+/Caspase+, respectively).

To quantify the fluorescence intensity at the axon initial segment (AIS), images were acquired with a Nikon AX R confocal microscope using a 60x-objective and resolution in Z-stack with 0.3 μm steps. To analyze stack images, a Matlab script freely available at: www.mathworks.com/matlabcentral/fileexchange/28181-ais-quantification was used as previously described [76, 77]. Briefly, a line profile was drawn along the fluorescently labeled AIS from the soma through and 5 μm past the AIS. Pixel fluorescence intensity values were averaged over a 3 × 3 pixel square centered on an arbitrarily drawn line, which was then smoothened using a 40-point sliding mean and normalized between 1 and 0 (maximum and minimum fluorescence intensity). The maximum position of the AIS was determined at the peak of the smoothed and normalized profile of fluorescence intensity. The start and end positions of the AIS were the proximal and distal sites, respectively, at which the profile dropped to 33% of its peak. To quantify the total number of synapses, confocal images were acquired at 60×, Synapsin-positive puncta with areas of 0.1–2 µm^2^ were considered bona fide synaptic boutons and automatically counted using ImageJ [78].

In SypHy experiments, to quantify the mean fluorescent changes upon KCl stimulation we manually designed the Region of Interest (ROI) of approximately 3 µm^2^ along responsive neurites and measured fluorescence signal in the average images obtained by leveraging the 15 images acquired in the different experimental conditions. An additional ROI was designed on the background to subtract background fluorescence from the measured values. The images were randomized using a computer-generated random sequence. Subsequently, two independent investigators, blinded to the group allocation of the randomized images, carried out the analysis.

### Proteomics sample processing

Cells from the different experimental classes were lysed, reduced, and alkylated with LYSE buffer (50 µL; Preomics) at 95 °C for 10 min and sonicated (3 cycles, 30 s/cycle) with an Ultrasonic Processor UP200St (Hielscher). The protein amount was assayed by the tryptophan method [79], and 10 μg of each sample was used. Proteins were isolated and digested by the protein aggregation capture method, automated on a KingFisher™ Apex robot (Thermo Fisher Scientific) in 96-well format [80]. Briefly, the tip plate was stored in plate #1. Lysate samples were stored in plate #2, at a final concentration of 70% acetonitrile, and with magnetic beads in a protein/bead ratio of 1:4 w/w (1:1 w/w SpeedBead magnetic carboxylate 45152105050250 and 65152105050250, Cytiva). Washing solutions were in plates #3– 5 (acetonitrile), plate #6 (70% ethanol), and plate #7 (isopropanol). Plate #8 contained 100 μl of digestion solution, namely 25 mM Tris HCl pH 8, endoproteinase LysC (Wako) in an enzyme/protein ratio of 1:100 w/w, and trypsin (Promega) in an enzyme/protein ratio of 1:50 w/w. Protein aggregation was carried out in two steps, each composed of a 1-min mixing at medium speed followed by a 10-min pause. Sequential washes were performed in 2.5 min at slow speed, without releasing the beads from the magnet. Digestion was set to 4 h, at 37 °C and at slow speed.

Obtained peptides were desalted in StageTips [81] and analyzed by a nano-UHPLC-MS/MS system using an Ultimate 3000 RSLC coupled to an Orbitrap Q Exactive Plus mass spectrometer (Thermo Scientific). Elution was performed with an Aurora Ultimate XT C18 UHPLC column (25 cm × 75 μm, 1.7 µm particle size, IonOpticks) at a flow rate of 300 nl/min, using a nonlinear gradient of 2–37% solution B (80% acetonitrile, 5% dimethyl sulfoxide, 0.1% formic acid in water) in 50 min. Orbitrap detection was used for MS1 measurements, at a resolving power of 70,000 in a range between 375 and 1500 *m*/*z*, and with a 3 × 10^6^ automatic-gain-control (AGC) target and 50 ms maximum injection time. Precursors were selected for data-independent fragmentation, with an isolation window width of 34 *m*/*z* and 19 loop count. Higher collisional dissociation energy was set to 27%, and MS2 scans were acquired at a resolution of 35,000, with a 3 × 10^6^ AGC target and 50 ms maximum injection time.

### Proteomics data analysis

All Data independent acquisition (DIA) raw files were processed with Spectronaut version 18 [82] using a library-free approach (direct DIA) under default settings. Enzymes/Cleavage Rules was set to Trypsin/P, LysC. Library was generated against UniProt Human (UP000005640_9606, 104602 entries) and Rattus norvegicus (UP000002494_10116, 31557 entries) databases. Carbamidomethylation was selected as a fixed modification, whereas methionine oxidation, N-terminal acetylation and deamidation (NQ) were selected as variable modifications. Significance thresholds for false discovery rates (FDRs) of peptide-spectrum matches and peptide/protein groups were set to 0.01. Precursor PEP Cutoff, Protein Qvalue Cutoff (Run) and Protein PEP Cutoff were set to 0.01. For quantification, Precursor Filtering was set to Identified (Qvalue), and MS2 was chosen as quantity MS-level. The mass spectrometry proteomics data have been deposited to the ProteomeXchange Consortium via the PRIDE [83] partner repository with the dataset identifier PXD070249. Proteomic analyzes were performed on two independent hiPSC lines, each evaluated across eight experimental conditions (iPSC, NPC_DIV 3, NPC_BrainPhys_DIV 7, NPC_Neurobasal_DIV 7, iGluNeuron HD NB DIV 42, iGluNeuron LD NB DIV49, iGluNeuron LD BP DIV 42, and iGluNeuron HD BP DIV 21), with four biological replicates per condition, resulting in a total of 64 samples. For coating conditions, Geltrex was used for iPSC and NPC_DIV3 samples, whereas all other conditions were cultured on poly-L-ornithine followed by laminin, as described previously.

### Taxonomic-based peptide annotation

To increase the accuracy of differential abundance analysis, filtering of human-specific peptides from human-rat co-culture samples was carried out by a twofold taxonomic-based peptide annotation, respectively involving Spectronaut and Proteoclade software. Proteoclade peptide annotation pipeline [84] was specified as follows: human proteome, encompassing a total of 20,421 exclusively reviewed sequences, was downloaded from UniProt database (code 9606) in fasta format, as well as the reviewed proteome of Rattus norvegicus species (code 10116) for a total of 92,906 entries. Consequently, downloaded fastas were merged by means of the utility “proteoclade.pcdb.merge_fastas” and the corresponding sequences in silico digested through the “proteoclade.pcdb.create_pcdb” function. The in silico digestion criteria to define the peptide database were specified to default settings, to closely resemble the experimental conditions, and included: “trypsin/p” protease cut rule, max number the protease is allowed to miss a cut set to 2, Excision of N-terminal methionines, a minimum and a maximum peptide database inclusion length, respectively of 7 and 55.

Lastly, quantified peptides were annotated by the “annotate_peptides” module, adopting the newly created Proteoclade database and the NCBI taxonomy. Proteoclade and Spectronaut peptide annotation tables were then merged and peptides filtered based on the following two criteria, which were deemed the best compromise between annotation sensitivity and specificity: in particular, peptides quantified on at least one rat astrocyte mono-culture sample were discarded, as well as peptides exclusively displaying a Rattus norvegicus annotation in spite of not being quantified on rat astrocyte mono-culture samples.

### Differential protein abundance analysis

A total of 104,584 peptides not meeting the algorithm selection criteria were discarded, along with 728 not-annotated peptides. The taxonomic-based filter yielded a peptide quantification matrix consisting of 104,440 human-specific peptides, which were consequently rolled up to 8869 unique protein features. A prevalence of 36% non-detects was observed across the 64 samples included in the proDA analysis. Consequently, the rolled-up protein count matrix was input to the differential abundance analysis pipeline (proDA), integrating the probabilistic dropout modeling of non-detect observations, which common high prevalence and a non-random nature in mass spectrometry quantification data could bias inferential tests. Specifically, raw protein counts were transformed through median normalization, a conservative normalization technique robust to non-detect observations. The Pearson correlation structure of the data was inspected to identify and exclude potential sample outliers lying below a mean correlation threshold of 0.8. Consequently, the quality-filtered and normalized protein count matrix was input to the software “proDA” function, which performed the probabilistic dropout and linear models fitting to the dataset. In particular, for the latter aim, the interpolated variables included: phenotype, culture medium, density metadata, and cell line metadata. The former three variables were conveniently merged, in order to test eight contrasts, through “test-diff” function, between model coefficients of interest: the first four, included iGluNeuron phenotypes across the four possible combinations of medium and density conditions against the hiPSC phenotype; the others tested coefficients across iGluNeurons culture conditions by focusing on medium comparison paired by culture density and vice versa. Sample cell line attribute was not deemed sufficiently informative, based on principal component analysis pattern recognition analysis (see Supplementary Fig. 4A) and due to its minor relevance with respect to the experimental design, it was not included in the coefficient contrast testing.

### Gene set enrichment analysis

The “clusterProfiler” R [85–88] package was used to perform the gene set enrichment analysis of differentially abundant proteins inferred by the proDA model.

For each of the 8 contrasts tested in the previous section, the complete set of differentially abundant proteins was sorted in decreasing order and tested for the enrichment in “Gene Ontology—Biological Process” terms, setting the significant threshold of adjusted *p*-value at 0.05. Specifically, the sets of ontologies significantly enriched for each iGluNeuron condition versus hiPSC phenotype contrast was filtered based on the following keywords: “axon”, “neuro”, “synaptic”. Additionally, filtered sets of enriched terms across iGluNeuron versus hiPSC contrasts were intersected to compare the enrichment score trend of common ontologies between the previous contrasts. On the other hand, the top 20 ontologies ranked by their negative enrichment score were selected from each set of enriched terms owing to a specific iGluNeuron condition versus hiPSC contrast. Lastly, each of the enriched ontology sets related to contrasts between iGluNeuron conditions was filtered for the aforementioned nervous system-specific keywords.

### Statistical analysis

Statistical analyzes were carried out using Prism (GraphPad Software Inc). Typical sample sizes were chosen in accordance with previous publications and are similar to those generally employed in the field. We estimated the normal distribution of the data with the Kolmogorov–Smirnov normality test. For not normally distributed data, we implemented a non-parametric Kruskal–Wallis with Dunn’s correction test for multiple comparison and a Mann–Whitney with Benjiamini–Hochberg’s correction test for pair-wise comparison. For normally distributed data, we performed a One-way ANOVA with Tukey’s correction for multiple comparison and an unpaired *t*-test with Benjiamini–Hochberg’s correction test for pair-wise comparison. All tests were performed as two-sided. To establish statistically significant differences, *p*-values < 0.05 were considered significant. All *p*-values of the computed statistical comparisons, included the significant ones shown in the Figures, are reported in the Supplementary Table 1. Data presentation and definition of error bars are specified in the corresponding Figure legends.

## Supplementary information

Suppl Fig 1

Suppl Fig 2

Suppl Fig 3

Suppl Fig 4

suppl fig legends

Supplementary Table 1

Supplementary Table 2

Supplementary Table 3

Supplementary Table 4

Supplementary Table 5

## Data Availability

The data that support the findings of this study are available from the corresponding author upon reasonable request.

## Code availability

The code is available on the following GitHub page “https://github.com/ennionano/functional_roadmap_for_induced_glut-neurons.” and will be fully accessible upon request.
