# Supplementary material for: An electrophysiological and proteomics roadmap for human induced glutamatergic neurons: fine-tuning of culture conditions for pathophysiological studies
Source: Cell Death Discov. 2026 Jun 5;12:333. doi: 10.1038/s41420-026-03185-w (PMC13458295; doi:10.1038/s41420-026-03185-w)
Supplement: Supplementary file 1 [file 41420_2026_3185_MOESM5_ESM.docx]

**Figure legends: supplementary tables**

**Supplementary Table 1. (A)** **First section**: P-values of the comparison between PEI and PLO coatings. **Second section:** P-values of the comparison between BP HD and BP LD features. **Third section:** P-values of the comparison between NB HD and NB LD features. **Fourth section:** P-values of the comparison between BP HD and NB HD features. **Fifth section:** P-values of the comparison between BP LD and NB LD features. **(B):** P-values of the comparison between all conditions at their DIV max.

**Supplementary Table 2.** Table of significant enriched "GO Biological Process" ontologies related to gene set enrichment analysis carried out on LFC(Log Fold Change) sets of "iGluNeurons_BP_HD vs iPSC", "iGluNeurons_BP_LD vs iPSC", "iGluNeurons_NB_HD vs iPSC" and "iGluNeurons_NB_LD vs iPSC" contrasts. First table column stores the ontology description followed by the enrichment score column, the normalized enrichment score column and column of ontology enrichment significance in the form of adjusted p-value (sig. threshold sitting at 0.05); last, the "core_enrichment" and "core_enrichment_symbol" columns report term enriching associated  genes, repsectively by NCBI and HUGO symbol nomenclature.

**Supplementary Table 3.** Upregulated neuron-specific proteins identified in the comparison with hiPSCs were associated with corresponding neurological disorders through the OMIM database (https://omim.org/).

**Supplementary Table 4.** Table of significant enriched "GO Biological Process" ontologies related to gene set enrichment analysis carried out on LFC sets of "iGluNeurons_BP_LD vs iGluNeurons_BP_HD", "iGluNeurons_NB_HD vs iGluNeurons_BP_HD" and "iGluNeurons_NB_LD vs iGluNeurons_BP_LD" contrasts. First table column stores the ontology description followed by the enrichment score column, the normalized enrichment score column and column of ontology enrichment significance in the form of adjusted p-value (sig. threshold sitting at 0.05); last, the "core_enrichment" and "core_enrichment_symbol" columns report term enriching associated genes, repsectively by NCBI and HUGO symbol nomenclature.

**Supplementary Table 5.** A quantitative analysis of synaptic properties at both the single-cell and network levels.
