# Supplementary material for: An electrophysiological and proteomics roadmap for human induced glutamatergic neurons: fine-tuning of culture conditions for pathophysiological studies
Source: Cell Death Discov. 2026 Jun 5;12:333. doi: 10.1038/s41420-026-03185-w (PMC13458295; doi:10.1038/s41420-026-03185-w)
Supplement: Supplementary file 2 [file 41420_2026_3185_MOESM6_ESM.docx]

**Supplementary Table statistics**

| DIV | 7 | 14 | 21 | 28 | 35 | 42 | 49 | 56 |
| --- | --- | --- | --- | --- | --- | --- | --- | --- |
| BP HD_PEI vs PLO_ | 0.75116 | 0.80512 | 0.80512 | 0.80512 | 0.81238 | 0.75116 | 0.80512 | 0.80512 |
| BP LD_PEI vs PLO_ | 0.62 | 0.78201 | 0.48436 | 0.62 | 0.78201 | 0.78201 | 0.78201 | 0.62 |
| NB HD_PEI vs PLO_ | 0.36836 | 0.75122 | 0.36836 | 0.47909 | 0.36836 | 0.61668 | 0.36836 | 0.36836 |
| NB LD_PEI vs PLO_ | 0.31076 | 0.38838 | 0.44539 | 0.00046624 | 0.0016893 | 0.04673 | 0.0027155 | 0.38838 |
| AE_BP HD vs BP LD_ | 3.0253e-05 | 8.4694e-09 | 7.9841e-15 | 0.8253 | 0.068536 | 1.7064e-05 | 1.0374e-08 | 0.01677 |
| MFR_BP HD vs BP LD_ | 5.588e-05 | 0.0013068 | 5.0903e-05 | 0.001404 | 5.0903e-05 | 4.078e-08 | 2.0085e-11 | 0.00017153 |
| MBR_BP HD vs BP LD_ | / | 2.3758e-05 | 0.83715 | 0.80098 | 0.086909 | 0.0037129 | 4.2859e-07 | 6.3338e-05 |
| RS_BP HD vs BP LD_ | / | 0.026779 | 0.78458 | 0.00057857 | 0.01662 | 0.14192 | 1.0124e-07 | 1.8586e-07 |
| NBR_BP HD vs BP LD_ | / | 0.0060657 | 0.0052081 | 0.38186 | 0.46285 | 0.0028601 | 3.8849e-07 | 0.00040261 |
| AE_NB HD vs NB LD_ | 0.0085506 | 4.8237e-06 | 2.3686e-10 | 4.577e-06 | 6.9773e-07 | 1.6173e-06 | 1.4299e-06 | 7.2612e-06 |
| MFR_NB HD vs NB LD_ | 0.12585 | 0.0041209 | 1.763e-06 | 0.10457 | 0.051339 | 3.2682e-09 | 0.019898 | 0.00084868 |
| MBR_NB HD vs NB LD_ | / | / | 1.2254e-07 | 0.0003467 | 0.92473 | 0.0049012 | 0.020573 | 0.0049012 |
| RS_NB HD vs NB LD_ | / | 0.18083 | 0.040038 | 0.60141 | 0.18083 | 0.00011633 | 0.00031248 | 0.0021981 |
| NBR_NB HD vs NB LD_ | / | / | 0.90334 | 0.081547 | 0.3415 | 0.081547 | 0.00015625 | 0.0016493 |
| AE_BP HD vs NB HD_ | 0.00016683 | 3.0748e-10 | 0.17874 | 3.1669e-05 | 1.0871e-07 | 6.0648e-12 | 1.0246e-13 | 6.7024e-13 |
| MFR_BP HD vs NB HD_ | 1.6111e-05 | 4.8014e-10 | 0.001114 | 0.12091 | 0.00045884 | 4.3184e-26 | 8.904e-16 | 1.093e-09 |
| MBR_BP HD vs NB HD_ | / | 2.7148e-12 | 0.25879 | 0.53389 | 0.42789 | 2.0476e-12 | 1.6466e-10 | 8.4895e-09 |
| RS_BP HD vs NB HD_ | / | 4.2257e-12 | 0.00059423 | 1.9465e-05 | 0.00098861 | 0.16576 | 2.5304e-09 | 2.5304e-09 |
| NBR_BP HD vs NB HD_ | / | 0.00038576 | 6.6754e-05 | 0.00029036 | 0.060758 | 5.9381e-06 | 6.6572e-10 | 3.2059e-09 |
| AE_BP LD vs NB LD_ | / | / | 0.82048 | 0.82048 | 0.82048 | 0.82048 | 0.017315 | 3.1178e-09 |
| MFR_BP LD vs NB LD_ | 0.207 | 0.00015651 | 0.00014663 | 0.19541 | 0.40989 | 0.0014723 | 0.5402 | 0.084421 |
| MBR_BP LD vs NB LD_ | / | 3.4556e-09 | 7.7732e-11 | 3.3474e-05 | 0.2657 | 0.078612 | 7.1191e-07 | 2.5134e-06 |
| RS_BP LD vs NB LD_ | / | 4.7418e-11 | 2.5546e-09 | 0.037033 | 0.99654 | 0.0014614 | 0.0035966 | 0.535 |
| NBR_BP LD vs NB LD_ | / | / | 0.051011 | 0.0017366 | 0.023926 | 0.4374 | 3.5127e-09 | 6.1163e-07 |

**Supplementary Table 1A:** **First section**: P-values of the comparison between PEI and PLO coatings. **Second section:** P-values of the comparison between BP HD and BP LD features. **Third section:** P-values of the comparison between NB HD and NB LD features. **Fourth section:** P-values of the comparison between BP HD and NB HD features. **Fifth section:** P-values of the comparison between BP LD and NB LD features.

| DIV | BP HD vs BP LD | BP HD vs NB HD | BP HD vs NB LD | BP LD vs NB HD | BP LD vs NB LD | NB HD vs NB LD |
| --- | --- | --- | --- | --- | --- | --- |
| MFR | 0.0007 | <0.0001 | <0.0001 | 0.287 | >0.9999 | 0.057 |
| MBR | 0.4279 | 0.0001 | 0.0461 | <0.0001 | <0.0001 | 0.1863 |
| BD | 0.5595 | 0.0513 | <0.0001 | <0.0001 | <0.0001 | 0.3755 |
| RS | >0.9999 | >0.9999 | <0.0001 | >0.9999 | <0.0001 | <0.0001 |
| MFIB | >0.9999 | 0.0017 | <0.0001 | 0.0192 | <0.0001 | 0.3976 |
| NBR | >0.9999 | 0.0151 | <0.0001 | 0.1147 | <0.0001 | 0.3122 |
| NBD | >0.9999 | 0.9115 | <0.0001 | 0.5219 | <0.0001 | 0.0002 |
| STH_peak_ | >0.9999 | 0.0368 | 0.0084 | >0.9999 | 0.5843 | >0.9999 |

**Supplementary Table 1B:** P-values of the comparison between all conditions at their DIV max.
